# Awareness and knowledge of sexually transmitted diseases (STDs) among school-going adolescents in Europe: a systematic review of published literature

**DOI:** 10.1186/1471-2458-11-727

**Published:** 2011-09-25

**Authors:** Florence N Samkange-Zeeb, Lena Spallek, Hajo Zeeb

**Affiliations:** 1 Bremen Institute for Prevention Research and Social Medicine, University of Bremen, Germany

## Abstract

**Background:**

Sexually transmitted diseases (STDs) are a major health problem affecting mostly young people, not only in developing, but also in developed countries.

We conducted this systematic review to determine awareness and knowledge of school-going male and female adolescents in Europe of STDs and if possible, how they perceive their own risk of contracting an STD. Results of this review can help point out areas where STD risk communication for adolescents needs to be improved.

**Methods:**

Using various combinations of the terms "STD", "HIV", "HPV", "Chlamydia", "Syphilis", "Gonorrhoea", "herpes", "hepatitis B", "knowledge", "awareness", and "adolescents", we searched for literature published in the PubMed database from 01.01.1990 up to 31.12.2010. Studies were selected if they reported on the awareness and/or knowledge of one or more STD among school-attending adolescents in a European country and were published in English or German. Reference lists of selected publications were screened for further publications of interest. Information from included studies was systematically extracted and evaluated.

**Results:**

A total of 15 studies were included in the review. All were cross-sectional surveys conducted among school-attending adolescents aged 13 to 20 years. Generally, awareness and knowledge varied among the adolescents depending on gender.

Six STDs were focussed on in the studies included in the review, with awareness and knowledge being assessed in depth mainly for HIV/AIDS and HPV, and to some extent for chlamydia. For syphilis, gonorrhoea and herpes only awareness was assessed. Awareness was generally high for HIV/AIDS (above 90%) and low for HPV (range 5.4%-66%). Despite knowing that use of condoms helps protect against contracting an STD, some adolescents still regard condoms primarily as an interim method of contraception before using the pill.

**Conclusion:**

In general, the studies reported low levels of awareness and knowledge of sexually transmitted diseases, with the exception of HIV/AIDS. Although, as shown by some of the findings on condom use, knowledge does not always translate into behaviour change, adolescents' sex education is important for STD prevention, and the school setting plays an important role. Beyond HIV/AIDS, attention should be paid to infections such as chlamydia, gonorrhoea and syphilis.

## Background

Sexually transmitted diseases (STDs) are a major health problem affecting mostly young people, not only in developing, but also in developed countries.

Over the period 1985-1996, a general decrease of gonorrhoea, syphilis and chlamydia infections was noted in developed countries, both in the general population and among adolescents [1]. From the mid-1990s however, increases in the diagnoses of sexually transmitted diseases, in particular syphilis, gonorrhoea and chlamydia have been reported in several European countries, especially among teenagers 16-19 years old [2-7].

The problem with most STDs is that they can occur symptom-free and can thus be passed on unaware during unprotected sexual intercourse. On an individual level, complications can include pelvic inflammatory diseases and possibly lead to ectopic pregnancies and infertility [8-11]. Female adolescents are likely to have a higher risk of contracting an STD than their male counterparts as their partners are generally older and hence more likely to be infected [2,12].

The declining age of first sexual intercourse has been proffered as one possible explanation for the increase in numbers of STDs [7]. According to data from different European countries, the average age of first sexual intercourse has decreased over the last three decades, with increasing proportions of adolescents reporting sexual activity before the age of 16 years [13-18]. An early onset of sexual activity not only increases the probability of having various sexual partners, it also increases the chances of contracting a sexually transmitted infection [19]. The risk is higher for female adolescents as their cervical anatomic development is incomplete and especially vulnerable to infection by certain sexually transmitted pathogens [20-23].

The reluctance of adolescents to use condoms is another possible explanation for the increase in STDs. Some surveys of adolescents have reported that condoms were found to be difficult to use for sexually inexperienced, detract from sensual pleasure and also embarrassing to suggest [24-26]. Condoms have also been reported to be used primarily as a protection against pregnancy, not STD, with their use becoming irregular when other contraceptives are used [15,27]. Furthermore, many adolescents do not perceive themselves to be at risk of contracting an STD [27].

We conducted this systematic review in order to determine awareness and knowledge of school-going adolescents in Europe of sexually transmitted diseases, not only concerning HIV/AIDS, but also other STDs such as chlamydia, gonorrhoea, syphilis and human papillomavirus (HPV). Where possible we will identify differences in awareness and knowledge by key demographic variables such as age and gender, and how awareness has changed over time.

Although knowledge and awareness have been reported to have a limited effect on changing attitudes and behaviour, [16,28-30] they are important components of sex education which help promote informed, healthy choices [31-33]. As schooling in Europe is generally compulsory at least up to the age of 15 years [34] and sex education is part of the school curriculum in almost all European countries, school-going adolescents should be well informed on the health risks associated with sexual activity and on how to protect themselves and others. In view of the decreasing age of sexual debut and the reported increasing numbers of diagnosed STDs among young people, results of our review can help point out areas where STD risk communication for school-attending adolescents needs to be improved.

## Methods

### Search strategy

We performed literature searches in PubMed using various combinations of the search terms "STD", "HIV", "HPV", "chlamydia", "syphilis", "gonorrhoea", "herpes", "hepatitis B", "knowledge", "awareness", and "adolescents". The reference lists of selected publications were perused for further publications of interest. The search was done to include articles published from 01.01.1990 up to 31.12.2010. Inclusion and exclusion criteria were specified in advance and documented in a protocol (Additional File 1).

### Inclusion criteria

Studies were selected if they reported on awareness and/or knowledge of one or more sexually transmitted disease(s) among school-attending adolescents in a European country, or in Europe as a whole, and were published in English or German.

### Exclusion criteria

Case reports, reviews, editorials, letters to the editor, expert opinions, studies on sexual activity/behaviour only, studies evaluating intervention programmes and studies not specifically on school-attending adolescents were excluded.

### Methodological assessment of reviewed studies

We used a modified version of the Critical Appraisal Form from the Stanford School of Medicine to assess the methodology of the studies included in the review [35]. The studies were classified according to whether or not they fulfilled given criteria such as 'Were the study outcomes to be measured clearly defined?', 'Was the study sample clearly defined?', or 'Is it clear how data were collected?' (Table 1). No points were allocated. Instead, the following categorisations could be selected for each assessment statement: 'Yes', 'Substandard', 'No', 'Not Clear', 'Not Reported', 'Partially Reported', 'Not Applicable', 'Not Possible to Assess', 'Partly'. The assessment was done independently by two of the authors (FSZ, LS) who then discussed their findings.

**Table 1:** Results of methodological assessment of studies included in the review

	Number of studies in each assessment category*								
	
Criteria	Y	S	N	NC	NR	PR	NA	NP	P
Did the study address a clearly focused issue?	15								

Was/were the study outcome(s) to be measured clearly described?	15								

Were the questions posed to assess outcome(s) clearly defined?	14		1						

Was the study samle clearly defined?	13								2

Were participating schools recruited in an acceptable way?	4	1		1			1	8	

Were the pupils recruited in an acceptable way? ^1^	11			4					

Were characteristics of subjects at enrolment reported?	12		1						2

Is it clear how data were collected?	15								

Did the authors mention that the instrument used for data collection was pre-tested or validated?	8		6						1

Were the questions posed appropriate to address given outcomes?	10			1				4	

Was participation rate reported?	9			2	4				

Was participation rate sufficiently high?	7	1	1					6	

Was the data analysis sufficiently rigorous?	15								

Were other factors accounted for that could affect outcomes?^2^	15								

Were results appropriately reported? ^3^	11	1							3

Is there a clear statement of findings?	15								

*Y = Yes, S = Substandard, N = No, NC = Not Clear, NR = Not Reported, PR = Partially Reported, NA = Not Applicable, NP = Not Possible to Assess, P = Partly

^1 ^did all pupils at the school(s), respectively in the grade concerned, have the same chance to participate?

^2 ^for example, sex, age, grade, school type, social class

^3 ^were numbers of outcome events reported on?

### Definition of awareness and knowledge

For the purpose of this review studies were said to have assessed awareness if participants were merely required to identify an STD from a given list or name an STD in response to an open question. Knowledge assessment was when further questions such as on modes of transmission and protection were posed.

## Results

**Figure 1:**
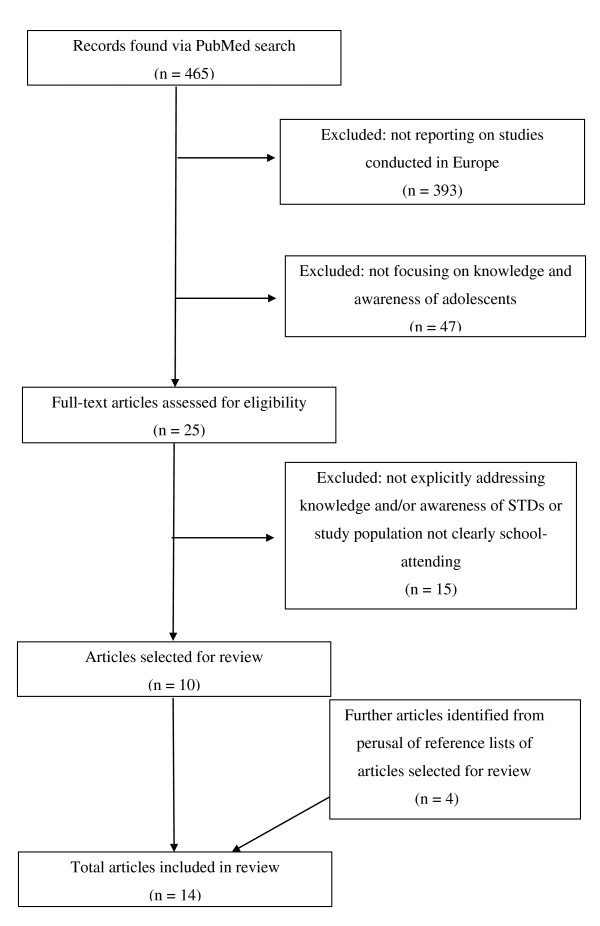
**Flow diagram showing selection process of articles included in the review**.

Overall, 465 titles and abstracts were obtained from the searches conducted. Three hundred and ninety-three articles were excluded as they did not report on studies conducted in Europe (Figure 1). A further 47 were excluded as they did not focus on knowledge and awareness of adolescents. Of the 25 identified articles dealing with knowledge on STDs among adolescents in Europe, 8 were excluded as they either did not specifically address the question of knowledge and/or awareness, or focused more on sexual behaviour/beliefs. A further seven articles were excluded because the study population was not clearly stated to be school-attending.

A review of the references listed in the 10 articles meeting inclusion criteria yielded four additional relevant articles. One article reported on two studies, hence a total of 15 studies published from 1990-2000 were included in the systematic review.

Six of the articles were published before the year 2000 [36-41], and nine after 2000 [42-49]. The studies report on surveys conducted from as early as 1986 to 2005 (Table 2).

**Table 2:** Characteristics of the 15 studies on knowledge on sexually transmitted diseases among school-attending adolescents in Europe

Reference	Study region, country	Year of study conduct	Survey instrument	Reported outcome(s) measured	Age of participants	No. of participants	Gender	Recruitment of pupils	Response rate
Fogarty [36]	Galway, Ireland	Not available	Classroom-completed questionnaire	Knowledge about AIDS	15-18 years	2614 leaving certificate pupils	Male/female	All 50 Galway second-level schools	Not available

Andersson-Ellström et al. [37]	Not specified, Sweden	1986 and 1988	Classroom-completed questionnaire	Knowledge of and attitudes towards STDs	18-19 years	1986: 350 1988: 603 upper secondary school pupils	Male/female	Not clear how many schools participated	100%

Tyden et al. [38]	Uppsala, Sweden	1988	Classroom-completed questionnaire	Knowledge of STDs and attitudes to condom	16-19 years	209 year 1 of upper secondary school pupils	Male/female	5 of 6 upper secondary schools	98%

Lunin et al. [39]	St. Petersburg, Russia	1993	Classroom-completed questionnaire	Knowledge, attitudes and behaviour relevant to AIDS prevention	14-17 years	370 year 10 pupils	Male/female	14 randomly selected schools	94%

Andersson-Ellström et al. [40]	Karlstad, Sweden	1989-1990	Questionnaire completed at clinic	Relationship between knowledge about STD, sexual behaviour, contraceptive use, STD protection and social class	16-18 years	88 year 1 of upper secondary school pupils	Female	Not clear how many schools participated	58%

Eriksson et al.[41]*	Not specified, Sweden	1994	Classroom-completed questionnaire	Knowledge on HIV/AIDS and sources of information	14-16 years	146 year 9 pupils	Male/female	1 school	100%

Garside et al. [42]**	Devon, England	1999-2000	Classroom-completed questionnaire	Knowledge and attitudes towards STDs, their detection and treatment	13-16 years	432 year 9 and 11 pupils	Male/female	1 school	Not reported

Goodwin et al. [43]^1^	St. Petersburg, Russia;	2000	Face-face interview in school	Knowledge on HIV/AIDS, sexual behaviour	Mean age 15.6 years	50 school pupils	Male/female	Not clear how many schools participated	Not clear

Goodwin et al. [43]^2^	St. Petersburg, Russia; Tblisi, Georgia; Kiev, Ukraine	Not available	Face-face interview in school and classroom-completed questionnaire	Knowledge on HIV/AIDS, sexual behaviour	14-17 years	102 school pupils	Male/female	Not clear how many schools participated	Not clear

Macek et al. [44]	Nova Gradiska/Zagreb, Croatia	Not available	Classroom-completed questionnaire	Knowledge on HIV/AIDS, attitudes towards integration of HIV-positive pupils into regular schools	Not available	108 year 7 and 8 pupils	Male/female	2 schools	Not reported

Woodhall et al.[45]***	Tampere, Finland	2005	Home-completed questionnaire	Knowledge of and attitudes towards STDs, esp. HPV	14-15 years	397 year 9 pupils	Male/female	All households in Tampere with adolescents born in 1990 and in year 9 contacted	21.5%

Gottvall et al. [46]	Not specific, Sweden	2008	Classroom-completed questionnaire	Knowledge of and attitudes towards HPV vaccination and condom use	15-16 years	608 year 1 of upper secondary school pupils	Male/female	7 schools	86%

Höglund et al. [47]	Uppsala, Sweden	Not available	Classroom-completed questionnaire	Knowledge of and attitudes to STDs, focus on HPV	15-20 years	459 year 1 of upper secondary school pupils	Male/female	5 schools	98%

Pelucchi et al.[48]***	Milan area and Varese, Italy	2008	Home-completed questionnaire	Knowledge of HPV, prevention, and attitudes towards vaccination	14-20 years	863 high school pupils	Male/female	8 schools	79%

Sachsenweger et al.[49]^#^	Mecklenburg-Western Pomerania, Germany	2005	Classroom-completed questionnaire	Knowledge on HIV/AIDS	11-18 years	769 year 7-9 pupils	Male/female	Not clear how many schools participated	Not available

* Other part of study conducted in Kenya. Only Swedish part reported on and included in this review

** Focus group discussions also held with local teenagers. Only details pertaining to questionnaire survey reported on and included in this review

^1,2 ^Publication reported on two separate studies. In both school children were compared to shelter children. Only details pertaining to school children are reported on and included in this review

*** Questionnaires also sent to parents. Only details pertaining to adolescents reported on and included in this review

^#^Publication in German

The majority of the 15 studies specifically focused on HIV/AIDS only (7 studies) [36,39,41,43,44,49], four on STDs in general [37,38,40,42], one on STDs in general with focus on HPV [47], and three on HPV only [45,46,48]. All the HPV studies were published after the approval and market introduction of the HPV vaccine in 2006.

Generally the studies were conducted in particular regions/towns in different countries, with only one being conducted across three towns in three different countries (Russia, Georgia and the Ukraine) [43]. Six of the studies were conducted in Sweden [37,38,40,41,46,47] two in Russia [39,43] and one each in Ireland, [36] England, [42] Croatia, [44] Finland, [45] Italy [48] and Germany [49] (Table 2).

In the studies, generally both male and female adolescents varying in age from 13-20 years were surveyed. One study surveyed females only [40] and adolescents 11-12 years old were included in only one study [49] (Table 2). Whereas most of the studies included assessed awareness and knowledge among boys and girls separately, only one study [48] specifically assessed the association between age and awareness/knowledge.

### Methodological summary of studies included in the review

All studies included in the review were cross-sectional in design. Apart from one study which recruited pupils by mailing the questionnaire to all households with adolescents in the 9^th ^grade, [45] pupils were recruited via schools. For 8 of the 15 studies it could not be deduced from the methods section how the participating schools were selected and in 4 studies it was not clear how the participating pupils were selected. The pupils completed questionnaires in school in 10 studies, and in two the questionnaires were completed at home [45,48]. Face-to-face interviews were used only in the surveys by Andersson-Ellström et al. [40] and by Goodwin et al. [43] (Table 2).

The study outcomes were clearly defined in all studies and the topics on which questions were posed were clearly described in all but one study. The majority of the studies also reported the individual questions posed to assess the given outcomes. In six studies the authors did not mention whether the instruments used for data collection had been pre-tested, validated, or whether the questions posed had been used in previous surveys (Table 1). Of the 9 studies which clearly reported participation rates, 7 had participation rates ranging from 79% to 100%. The remaining two studies had participation rates of 21.5% and 58% (Table 2).

Six STDs were focussed on in the studies included in the review, with awareness and knowledge being assessed in depth mainly for HIV/AIDS and HPV,[36,41-43,46-49] and to some extent for chlamydia [37,38,42,47]. For syphilis, gonorrhoea and herpes, only awareness was assessed in four studies [37,38,42,47].

### Awareness and knowledge of HPV

The reported awareness of HPV among the surveyed adolescents was generally low (identification from given list), ranging from 5.4% in the study by Höglund et al. [47] to 66% in the study by Pelucchi et al. [48]. In the two studies which also reported results for females and males separately, awareness was observed to be statistically significantly higher among females than among males: 16.4% vs. 9.6% in the Swedish study by Gottvall et al. [46] and 71.6% vs. 51.2% in the Italian study by Pelucchi et al. [48]. In the study by Höglund et al., only one of the participating 459 adolescents mentioned HPV (in response to an open question on known STDs) [47].

Awareness of the HPV vaccine was also very low, with 5.8% and 1.1% of adolescents surveyed in the studies by Gottvall et al. and Höglund et al. respectively, reporting being aware of the vaccine [46,47]. Whereas only 2.9% and 9.2% of adolescents in these two Swedish studies were aware that HPV is sexually transmitted, the proportion was 60.6% in the Italian study [48]. A minority of adolescents knew that HPV is a risk factor for cervical cancer: 1.2% in the study by Höglund et al. [47] and 8.1% in the study by Gottvall et al. [46]. Among the adolescents who participated in the survey by Pelucchi et al., 48.6% were aware that the aim of the HPV vaccine is to prevent cervical cancer [48]. Among female adolescents who participated in the study by Gottvall et al., 11.8% did not believe they would be infected with HPV [46]. The proportion was 55% among female participants in the study by Pelucchi et al. [48]. The latter study surveyed pupils aged 14-20 years but did not report on age differences in awareness.

Three studies reported on awareness of condylomata, genital warts which are caused by the human papilloma virus. Two of the studies reported awareness of 35% [38] and 43% [37]. The third study mentioned that awareness of condylomata was lower than that for chlamydia without stating the corresponding figures [40].

### Awareness and knowledge of HIV/AIDS

Knowledge and awareness was quite high in all studies reporting on HIV/AIDS, with more than 90% of adolescents being able to identify the disease as an STD from a given list or in response to the direct question "Have you ever heard of HIV/AIDS?" [36,38,42]. In one study where the open question "Which STDs do you know or have you heard of?" was used, 88% of respondents mentioned HIV/AIDS [47] (Table 3).

**Table 3:** Awareness and knowledge of STDs reported in 14 of the 15 studies included in the review

Question/Item assessed in studies	Females % (reference)	Males % (reference)	Females and males % (reference)
**HPV**			

Heard of HPV (identification from list of STDs or direct question, 'Have you heard of HPV?')	71.6% (Pelucchi et al.) [49] 16.4% (Gottvall et al.) [46]	51.2% (Pelucchi et al.) [49] 9.6% (Gottvall et al., 2009) [46]	66.6% (Pelucchi et al.)** [49] 13.5% (Gottvall et al.)** [46] 5.4% (Höglund et al.) [47] 33% (Woodhall et al.) [45]

Heard of HPV (open question - 'Which STDs do you know/have you heard of?')			0.2% (Höglund et al.) [47]

Heard of HPV vaccine	9.2% (Gottvall et al.) [46]	1.1% (Gottvall et al.) [46]	5.8% (Gottvall et al.)** [46] 1.1% (Höglund et al.) [47]

Participants who knew that HPV is sexually transmitted	64.9% (Pelucchi et al.) [49] 12.1% (Gottvall et al.) [46]	47.4% (Pelucchi et al.) [49] 5.4% (Gottvall et al.) [46]	60.6% (Pelucchi et al.) [49] 9.2% (Gottvall et al.)** [46] 2.9% (Höglund et al.) [47]

Participants who knew that HPV is a risk factor for cervical cancer (closed question)	11.8% (Gottvall et al.) [46]	3.1% (Gottvall et al.) [46]	8.1% (Gottvall et al.) [46] 1.2% (Höglund et al.) [47]

Participants aware that aim of HPV vaccination is to prevent cervical cancer	53.9% (Pelucchi et al.) [49]	32.1% (Pelucchi et al.) [49]	48.6% (Pelucchi et al.)** [49]

Participants who thought that aim of HPV vaccination is to prevent an STD	8.6% (Pelucchi et al.) [49]	7.2%(Pelucchi et al., 2010) [49]	8.3% (Pelucchi et al.) [49]

Subjective rating of risk of contracting HPV	45% perceived at risk (Pelucchi et al.) [49] 11.8% do not believe will be infected (Gottvall et al.) [46]	26% perceived at risk (Pelucchi et al.) [49] 24.5% do not believe will be infected (Gottvall et al.) [46]	17.3% do not believe will be infected (Gottvall et al.)** [46]

**HIV**			

Heard of HIV (identification from list of STDs or direct question)	97.2% among year 9 and 100% among year 11 pupils (Garside et al.) [42]	97.0% among year 9 and 96.2% among year 11 pupils (Garside et al.) [42]	97.7% (Garside et al.) [42] 100% (Tyden et al.) 91% [38] (Fogarty) [36]

Heard of HIV (open question - which STDs do you know/have you heard of?)			88% (Höglund et al.) [47]

Participants who knew that you can not tell by looking at someone if they have HIV			Overall 53% (Goodwin et al.)^2 ^[43]

Participants who knew that HIV is caused by a virus			91% (Eriksson et al.) [41] 88% (Fogarty) [36]

Participants who knew that HIV is sexually transmitted	99% (Sachsenweger et al.) ^# ^[49] 97% (Goodwin et al.)^1^[43]	99% (Sachsenweger et al.) ^#^[49] 86% (Goodwin et al.)^1^[43]	99% (Sachsenweger et al.) ^#^[49] 81% (Höglund et al.) [47] 92% (Goodwin et al.)^1^[43] 99% (Eriksson et al.) [41] 95% (Fogarty) [36]

Participants who knew that sharing a needle with an HIV infected person may lead to infection with the virus	91% (Sachsenweger et al.) ^#^[49] 72% (Goodwin et al.)^1^[43]	91% (Sachsenweger et al.) ^#^[49] 62% (Goodwin et al.)^1^[43]	91% (Sachsenweger et al.) ^#^[49] 68% (Goodwin et al.)^1^[43] 95% (Eriksson et al.) [41] 99% (Fogarty) [36]

Participants who knew that use of condoms can protect against contraction of HIV	83%(Goodwin et al.)^1^[43] 42% (Lunin et al.) [39]	86% (Goodwin et al.)^1^[43] 60% (Lunin et al.) [39]	99% (Sachsenweger et al.) ^#^[49] 76% (Höglund et al.) [47] 84% (Goodwin et al.)^1^[43] 99% (Eriksson et al.) [41] 51% (Lunin et al., 1995)** [39] 94% (Fogarty) [36]

Participants who knew where to go for diagnosis/treatment/advice on HIV			22% aware of STD clinic and 32% of AIDS telephone service (Fogarty) [36]

Subjective rating of risk of contracting HIV	11% perceived themselves 'not at risk' (Lunin et al.) [39]	19% perceived themselves 'not at risk' (Lunin et al.) [39]	15% perceived themselves 'not at risk' (Lunin et al.)** [39]

**Chlamydia**			

Heard of chlamydia (identification from list of STDs or direct question)	41.4% among year 9 and 22.7% among year 11 pupils (Garside et al.) [42] 79% (Tyden et al.) [38]	36.7% among year 9 and 13.2% among year 11 pupils (Garside et al.) [42] 60% (Tyden et al.) [38]	34% (Garside et al.)*** [42] 70% (Tyden et al.) [38] 91% in 1986, and 96% in 1988 survey (Andersson-Ellström et al.) [37]

Heard of chlamydia (open question - which STDs do you know/have you heard of?)			86% (Höglund et al.) [47]

Participants who knew that chlamydia can be symptom-free	51% in 1986, and 68% in 1988 survey (Andersson-Ellström et al.) [37]	28% in 1986, and 45% in 1988 survey (Andersson-Ellström et al.) [37]	46% (Höglund et al.) [47] 40% in 1986, and 56% in 1988 survey (Andersson-Ellström et al.)** [37]

**Syphilis**			

Heard of syphilis (identification from list of STDs or direct question)	45.5% among year 9 and 47.0% among year 11 pupils (Garside et al.) [42]	43.4% among year 9 and 45.3% among year 11 pupils (Garside et al.) [42]	45% (Garside et al.) [42]

**Gonorrhoea**			

Heard of gonorrhoea (identification from list of STDs or direct question)	51.0% among year 9 and 53.0% among year 11 pupils (Garside et al.) [42]	52.4% among year 9 and 60.4% among year 11 pupils (Garside et al.) [42]	53% (Garside et al.) [42] ≥ 84% (Tyden et al.) [38] 98%, 1986 survey (Andersson-Ellström et al.) [37]

Heard of gonorrhoea (open question - which STDs do you know/have you heard of?)			50% (Höglund et al.) [47]

**Herpes**			

Heard of herpes (identification from list of STDs or direct question)	52.4% among year 9 and 75.8% among year 11 pupils (Garside et al.) [42]	53.6% among year 9 and 71.7% among year 11 pupils (Garside et al.) [42]	59.1% (Garside et al.)*** [42] 90%, 1986 survey (Andersson-Ellström et al.) [37] 56% (Tyden et al.) [38]

Heard of herpes (open question - which STDs do you know/have you heard of?)			64% (Höglund et al.) [47]

**STDs in general**			

Participants who knew that STDs in general can be symptom-free	53.8% among year 9 and 60.0% among year 11 pupils (Garside et al.) [42]	64.2% among year 9 and 60.4% among year 11 pupils (Garside et al.) [42]	59.7% (Garside et al.) [42]

Participants who knew that use of condoms can protect against contraction of STDs in general	15%, 1986 survey (Andersson-Ellström et al.) [37] 34%, 1988 survey (Andersson-Ellström et al.) [37] 100% (Andersson-Ellström et al.) [40]	27%, 1986 survey (Andersson-Ellström et al.) [37] 52%, 1988 survey (Andersson-Ellström et al.) [37]	20%, 1986 survey (Andersson-Ellström et al.)** [37] 43%, 1988 survey (Andersson-Ellström et al.)** [37] 100% (Tyden et al.) [38]

Subjective rating of risk of contracting an STD in general	32%, 1986 survey (Andersson-Ellström et al., 1991) [37] 24%, 1988 survey (Andersson-Ellström et al.) [37]	16%, 1986 survey (Andersson-Ellström et al.) [37] 24%, 1988 survey (Andersson-Ellström et al.) [37]	55% "low" perceived susceptibility (Woodhall et al.)* [45]

**Reported use of condoms**			
Participants who reported using condoms at first sexual intercourse	50% (Tyden et al.) [38] 65% (Gottvall et al.) [46]	40% (Tyden et al.) [38] 65% (Gottvall et al.) [46]	45% (Tyden et al.) [38] 65% (Gottvall et al.) [46] 61% (Höglund et al.) [47]

Participants who reported using condoms at last sexual intercourse	26% (Tyden et al.) [38]	38% (Tyden et al.) [38]	31% (Tyden et al.) [38]

* combined figure given for HPV and chlamydia

** statistically significant differences in awareness/knowledge between boys and girls

*** statistically significant differences in awareness/knowledge between year 9 and year 11 pupils

^# ^Publication in German

In the studies where this was asked, a large majority of the adolescents knew that HIV is caused by a virus, [36,41] is sexually transmitted,[36,41,43,47,49] and that sharing a needle with an infected person may lead to infection with the virus [36,41,43,49]. Statistically significant age specific differences in knowledge on mode of HIV-transmission were reported in the study conducted in Germany [49]. Compared to 13 and 15 year old pupils, a higher proportion of 14 year old pupils correctly identified the level of risk of HIV-transmission associated with bleeding wounds, intravenous drug use and sexual contact. For the latter mode of transmission, the lowest proportion of correct answers was observed among 16 year old pupils. Generally the proportion of respondents correctly reporting that use of condoms helps protect against contraction of HIV was above 90%. The only exception was in the Russian study conducted by Lunin et al. in 1993, in which only 42% of females and 60% of males were aware of this fact [39]. In the same study, only 15% of the adolescents perceived themselves 'not at risk' of contracting HIV (Table 3).

Only one study reported asking the adolescents if one can tell by looking at someone if they have HIV, to which 47% responded affirmatively [43].

### Awareness and knowledge of chlamydia

The proportion of adolescents able to identify chlamydia as an STD from a list of diseases ranged from 34% in the study conducted in England by Garside et al. [42] to 96% in the Swedish study by Andersson-Ellström et al. [22]. In the Garside study, the proportion was higher among year 9 than among year 11 pupils (p < 0.05). In another Swedish study by Höglund et al. 86% of the surveyed adolescents mentioned chlamydia as one of the STDs known to them in response to an open question [47]. In the two studies which reported on awareness among boys and girls separately, girls were observed to have higher awareness proportions than boys [38,42]. While the observation was not statistically significant in one of the studies, [27] this was not reported on in the other study [38].

Not many adolescents knew that chlamydia can be symptom-free: 40% and 56% in the 1986 and 1988 surveys by Andersson-Ellström et al. [37] and 46% in the study by Höglund et al. [47]. In one Swedish study where the level of knowledge in the same study population was assessed at age 16 and 18, a statistically significant increase in knowledge was observed over time [40]. Only the Finish study reported on the subjective rating of risk of contracting chlamydia. 55% of the adolescents surveyed reported 'low perceived susceptibility' [45] (Table 3).

### Awareness and knowledge of gonorrhoea

Gonorrhoea was identified as an STD from a given list by 84% of adolescents in the survey by Tyden et al.,[38] by 98% in the survey by Andersson-Ellström et al.,[37] and by 53% in the survey by Garside et al. [42]. In the latter, the difference between year 9 and year 11 pupils was more pronounced among boys: 53% among year 9 and 60% among year 11 (p > 0.05). A statistically significant increase in knowledge over time was observed in a group of girls surveyed at age 16 and 18 [40]. Only 50% of the adolescents surveyed in the study by Höglund et al. mentioned gonorrhoea in response to an open question on known STDs [47] (Table 3).

### Awareness of syphilis and herpes

Awareness of syphilis was surveyed only in the study conducted in England where 45% of the participating adolescents correctly identified the disease from a given list as an STD. The proportion was slightly higher among year 11 compared to year 9 pupils and awareness was slightly higher among girls than among boys (p > 0.05) [42] (Table 3).

In the Tyden et al. study, [38] 56% of the surveyed adolescents identified herpes as an STD from a given list. The proportion was 90% in the survey by Andersson-Ellström et al. [37] and 59% in the Garside et al. study [42]. In the latter, considerable differences were observed between year 9 and year 11 pupils (p < 0.05), but not between girls and boys in the same school year. Herpes was mentioned as an STD by 64% of the adolescents surveyed in the study by Höglund et al. [47] (Table 3).

### Awareness of STDs in general

Five of the studies reviewed assessed the knowledge of participating adolescents on STDs in general. In the England study, all in all 59.7% of the participants knew that STDs in general can be symptom-free [42]. Among girls, knowledge was higher among year 11 than year 9 pupils, while the opposite was true for boys. The proportion of boys in year 9 who knew this fact (64.2%) was considerably higher than that of year 9 girls (53.8%) (Table 3). In two Swedish studies by Tyden et al. and by Andersson-Ellström et al., all surveyed adolescents knew that the use of condoms can protect against the contraction of STDs in general [38,40]. In an earlier study by Andersson-Ellström et al., 20% of sexually active pupils surveyed in 1986 were aware that condoms protect against infection. The figure significantly went up to 43% in 1988, with boys having significantly higher awareness than girls in both years [22] (Table 3). In the same study, the proportion of girls who felt themselves to be at risk of contracting an STD in general went down from 32% in the 1986 survey to 24% in the 1988 survey. Among boys, the proportion increased from 16% in 1986 to 24% in 1988. These changes were not statistically significant [37]. In the Finish study, 55% of the surveyed adolescents perceived themselves to be at low risk of contracting an STD [45].

### Reported use of condoms

Use of condoms by sexually active participants was assessed in three studies, all conducted in Sweden [38,46,47]. Reported use at sexual debut was lowest in the study published in 1991 (31%), [38] and higher in the other studies both published in 2009: 61% [47] and 65% [46] respectively (Table 3). In the earlier study, the proportion of girls reporting condom use was, at 50%, considerably higher than that of boys (40%) [38]. In the study by Gottvall et al., no difference in condom use was observed between girls and boys [46]. Condom use at recent coitus was reported on only in the earlier study [38]. It was observed that the decrease in the proportion of girls reporting using condoms was more pronounced than that of boys (26% vs. 40%) (Table 3).

## Discussion

The highest awareness and knowledge were reported for HIV/AIDS. This is certainly linked to the fact that since the mid 1980s, extensive awareness campaigns on this topic have been conducted globally. The lowest proportions were reported for HPV, with awareness as low as 5.4% in one study [47]. With only about 1 in 8 respondents knowing that HPV is an STD, awareness was still very low in one of the two studies conducted after the introduction of the HPV vaccine [46]. A higher awareness (66.6% of respondents aware), measured in a different population, was observed in the second recent study on HPV [48].

Two factors appeared to have influenced awareness. The first was of a methodological nature and related to the fact whether an open or closed question was posed. Of the studies included in the review which assessed awareness, all but one used closed-form questions only. The adolescents either had to identify sexually transmitted diseases from a given list of diseases, or the question was in a yes/no format. Initially, Höglund et al. asked participating adolescents to list all STDs known to them and then later on, if they had ever heard of HPV. Only one participant (0.2%) mentioned HPV as one of the STDs known to them, but later, 24 (5.4%) reported to have heard of HPV [47]. In comparison to open-form questions, closed questions are not only more practical and easier to respond to, but also easier to code and analyse. One of the arguments raised against closed questions, especially where a list of possible answers is given, is the risk of guesswork. It can not be ruled out that some participants, unable to answer the question, will select answers at random [50,51]. In the study by Garside et al. for example, among year 9 pupils, 14.5% incorrectly identified plasmodium, and 20.6% filariasis from a given list as STDs [42]. Open questions have been recommended for surveying participants with unknown or varying knowledge/awareness [50] as these questions provide a more valid picture of the state of knowledge [51].

To a lesser extent, gender also appears to have influenced knowledge and awareness, especially for HPV [46,48]. Significant gender differences were observed, with females having better awareness and knowledge than males. Although the data are limited as not all studies reported results separately for males and females, these findings, could be reflective of the way awareness campaigns, for example on HPV, have been targeted more at females than at males.

The studies on HIV included in our review generally reported high awareness of the protective effect of condoms among adolescents [36,41,43,47,49]. One study included in the review however observed that adolescents seem to regard condoms primarily as a method of contraception and not as a means of protection against sexually transmitted diseases (40). In this study, 19 out of 20 female adolescents who reported more than 4 sexual partners at the age of 18 reported intercourse without a condom in relationships of less than 6 months' duration. The majority of them were, however, convinced that they had neither acquired (96%) nor transmitted (93%) an STD at last unprotected intercourse [40]. Other studies also indicate that consistent condom use is generally low among adolescents [27,52-55].

Where reported, participation rates were generally high, probably due to the fact that the adolescents were recruited in schools. In some instances however, the number of participants was low even though the participation rate was reported as high. In the study by Tyden et al. for example, the study sample consisted of 213 pupils, 12% of the 1830 students in the first form of upper secondary school in Uppsala [38]. The authors base the participation rate of their study (98%) on the 12%, without explaining how it came about that only 213 pupils were considered for participation. The one study which recruited participants per post had a very low participation rate of 21.5% [45]. Nevertheless, the study had more participants than others with comparatively higher participation rates. Bias related to selective participation is an issue that needs to be considered on a study by study basis, and reporting on response proportions should be considered essential for all studies.

### Study strengths and limitations

To our knowledge no systematic reviews of published literature on knowledge and awareness of sexually transmitted diseases among school-attending adolescents in Europe have been conducted to date. The current review confirms that there are considerable gaps in knowledge and awareness on major STDs in European adolescents. Our results underline the importance of the objectives set for adolescents' sexual and reproductive health in Europe, the first of which foresees that adolescents be informed and educated on all aspects of sexuality and reproduction [31].

We could not identify many studies on knowledge and awareness of sexually transmitted diseases among school-attending adolescents in Europe. This could be due to the fact that knowledge has been shown to have little impact on behaviour change, and prevention interventions have generally moved away from a focus on knowledge and awareness as key mediators. Another possible reason is that schools are not always willing to participate in such studies due to competing demands of other school activities or because of the subject content [16,28-30].

One limitation of our review is that the 15 studies included did not all focus on the same sexually transmitted diseases. The four studies conducted in Eastern Europe were all on HIV/AIDS knowledge and awareness only, whereas Western European studies were on STDs in general or on HPV. Furthermore, the formulation of the questions used to assess awareness and knowledge varied between studies, making it difficult to directly compare the findings of individual studies. Another potential limiting factor is the age variation of participants in the studies included in the review, especially as all but one study did not clearly investigate the association between age and awareness or knowledge. Due to the afore-mentioned factors and the small number of studies available, it was not possible to perform a meta-analysis of the study findings.

The representativeness of study participants in some studies could not be assessed as it was not mentioned how the schools were selected [37,40-44,49]. Different socioeconomic environments of individual schools are likely to affect results, but there is currently not sufficient information to assess this.

The school setting offers an effective way to access adolescent populations universally, comprehensively and uniformly [56]. It plays an important role for sex education, especially for those adolescents with no other information sources. Furthermore, some parents are not comfortable discussing sexual issues with their children. It therefore comes as no surprise that many young people cite the school as an important source of information about sexually transmitted diseases [26,27]. Although sex education is part of the school curriculum in many European countries, there are differences in the issues focused on. In some countries sex education is integrated in life skills approach, whilst biological issues are predominant in others and at times the focus is on HIV/AIDS prevention [57]. Generally it seems that education schedules offer a range of opportunities to raise knowledge and awareness of STD among adolescents.

## Conclusion

In general, the studies reported similar low levels of knowledge and awareness of sexually transmitted diseases, with the exception of HIV/AIDS. Although, as shown by some of the findings on condom use, knowledge does not always translate into behaviour change, adolescents' sex education is important for STD prevention, and the school setting plays an important role. Beyond HIV/AIDS, attention should be paid to infections such as chlamydia, gonorrhoea and syphilis.

## Competing interests

The authors declare that they have no competing interests.

## Authors' contributions

FSZ developed the concept for the study, conducted the literature search, assessed studies for inclusion in the review and extracted data. She also prepared drafts and undertook edits. LS was involved in the development of the study concept, conducted the literature search, assessed studies for inclusion in the review and extracted data. HZ was involved in the development of the study concept. All authors contributed to the editing of the drafts and have read and approved all versions of the manuscript.

## Pre-publication history

The pre-publication history for this paper can be accessed here:

http://www.biomedcentral.com/1471-2458/11/727/prepub

## Supplementary Material

Additional file 1 **Review Protocol**: The preparation process for the systematic review is documented in the file. Included are the objectives of the review, inclusion and exclusion criteria, the search strategy, definition of outcomes, as well as the data abstraction table. Click here for file

## References

[B1] World Health Organisation Global prevalence and incidence of selected curable sexually transmitted infections 2001 WHO, Geneva

[B2] Panchaud C Singh S Feivelson D Darroch JE Sexually transmitted diseases among adolescents in developed countries Fam Plan Persp 2000 32 24 32 &45 10.2307/2648145 10710703

[B3] Berglund T Fredlund H Giesecke J Epidemiology of the re-emergence of gonorrhoea in Sweden Sex Transm Dis 2001 111 114 10.1097/00007435-200102000-00009 11234784

[B4] Health protection Surveillance Centre. Surveillance of STI A report by the Sexually Transmitted Infections subcommittee for the Scientific Advisory committee of the health Protection Surveillance Centre December 2005

[B5] Nicoll A Hamers FF Are trends in HIV, gonorrhoea and syphilis worsening in Western Europe? BMJ 2002 324 1324 1327 10.1136/bmj.324.7349.1324 12039830 PMC1123279

[B6] Twisselmann B Rising trends of HIV, gonorrhoea, and syphilis in Europe make case for introducing European surveillance systems Euro Surveill 2002 6 23 pii = 1952 http://www.eurosurveillance.org/ViewArticle.aspx?ArticleId=1952 Last accessed 30.11.2010

[B7] Adler MW Sexually transmitted infections in Europe Eurohealth 2006 12 3 6

[B8] PHLS, DHSS & PS and the Scottish ISD(D)5 Collaborative Group Trends in Sexually Transmitted Infections in the United Kingdom 1990-1999 2000 Public Health Laboratory Service London

[B9] Stamm W Guinan M Johnson C Effect of treatment regiments for Neisserie gonorrhoea on simultaneous infections with Chlamydia trachomatis New Eng J Med 1984 310 545 559 10.1056/NEJM198403013100901 6363935

[B10] MacDonald NE Brunham R The Effects of Undetected and Untreated Sexually Transmitted Diseases: Pelvic Inflammatory Disease and Ectopic Pregnancy in Canada The Canadian Journal of Human Sexuality 1997 6 2 Special Issue: STDs and Sexual/Reproductive Health

[B11] Simms I Stephenson JM Pelvic inflammatory disease epidemiology; What do we know and what do we need to know? Sex Trans Inf 2000 76 80 87 10.1136/sti.76.2.80 PMC1758284 10858707

[B12] Bozon M Kontula O M Hubert, N Bajos, and T Sandfort Sexual initiation and gender in Europe Sexual behavior and HIV/AIDS in Europe 1998 London: UCL Press 37 67

[B13] Kangas I Andersen B McGarrigle CA Ostergaad L A comparison of sexual behaviour and attitudes of healthy adolescents in a Danish high school in 1982, 1996 and 2001 Pop Health Metr 2004 http://www.pophealthmetrics.com/content/2/1/5 online publication: last accessed 03.12.2010 10.1186/1478-7954-2-5 PMC394347 15038827

[B14] Ross J Godeau E Dias S Currie C, Roberts C, Morgan A, et al Sexual health Young people's health in context. Health Behaviour in School-aged Children (HSBC) study: International report from the 2001/2002 survey 2004 Copenhagen: WHO21940727

[B15] Bundeszentrale für gesundheitliche Aufklärung Jugendsexualität. Repräsentative Wiederholungsbefragung von 14- bis 17-Jährigen Jugendlichen und ihren Eltern 2006 BZgA

[B16] Tucker JS Fitzmaurice AE Imamura M Penfold S Penney GC van Teijlingen E Schucksmith J Philip KL The effect of the national demonstration project *Healthy Respect *on teenage sexual health behaviour Eur J Public Health 2006 17 1 33 41 10.1093/eurpub/ckl044 16601108

[B17] Godeau E Gabhainn SN Vignes C Ross J Boyce W Todd J Contraceptive use by 15-year-old students at their last sexual intercourse: Results from 24 countries Arch Paediatr Adolesc Med 2008 162 66 73 10.1001/archpediatrics.2007.8 18180415

[B18] Bundeszentrale für gesundheitliche Aufklärung **Sexualität und Migration: Milieuspezifische Zugangswege für die Sexualaufklärung Jugendlicher. Ergebnisse einer repräsentativen Untersuchung der Lebenswelten von 14- bis 17-Jährigen Jugendlichen mit Migrationshintergrund** 2010 BZgA

[B19] Heinz M Sexuell übertragbare Krankheiten bei Jugendlichen: Epidemiologische Veränderungen und neue diagnostische Methoden 2001 Arbeitsgemeinschaft Kinder-und Jugendgynäkologie e.V http://www.kindergynaekologie.de/html/kora22.html last accessed 03.12.2010

[B20] Berrington de González A Sweetland S Green J Comparisons of risk factors for squamous cell and adenocarcinomas of the cervix: a meta-analysis Br J Cancer 2004 90 1787 1791 15150591 10.1038/sj.bjc.6601764 PMC2409738

[B21] Reich O Is early first intercourse a risk factor for cervical cancer? Gynäkol Geburtshilfliche Rundsch 2005 45 251 256 10.1159/000087143 16205092

[B22] Gille G Klapp C Chlamydia trachomatis infections in teenagers Der Hautarzt 2007 58 31 37 10.1007/s00105-006-1265-x 17165068

[B23] Hwang LY Ma Y Miller Benningfield S Clayton L Hanson EN Jay J Jonte J Godwin de Medina C Moscicki AB Factors that influence the rate of epithelial maturation in the cervix of healthy young women J Adolesc Health 2009 44 2 103 110 10.1016/j.jadohealth.2008.10.006 19167657 PMC2662755

[B24] Kegeles SM Adler NE Irwin CE Adolescents and condoms Am J Dis Child 1989 143 911 915 2756965

[B25] Ford N The AIDS awareness and sexual behaviour of young people in the South-west of England J Adolesc 1992 15 393 413 10.1016/0140-1971(92)90071-C 1487576

[B26] Persson E Sandströäm B Jarlbro G Sources of information, experiences and opinions on sexuality, contraception and STD protection among young Swedish students Advances in Contraception 1992 8 41 49 10.1007/BF01849347 1590101

[B27] Editorial team Young people's knowledge of sexually transmitted infections and condom use surveyed in England Euro Surveill 2005 10 31 pii = 2766 Last accessed 30.11.2010 16785677

[B28] Lister-Sharpe D Chapman S Stewart-Brown S Sowden A Health promoting schools amd health promotion in schools: two systematic reviews Health Technol Assess 1999 3 22 1 207 10683593

[B29] Wight D Raab G Henderson M Abraham C Buston K Hart G Scott S Limits of teacher delivered sex education: interim behavioural outcomes from randomised trial BMJ 2002 324 1 6 12065268 10.1136/bmj.324.7351.1430 PMC115856

[B30] Stephenson J Strange V Forrest S Oakley A Copas A Allen E Babiker A Black S Ali M Monteiro H Johnson AM Pupil-led sex education in England (RIPPLE study): cluster randomised intervention trial Lancet 2004 364 9431 338 346 10.1016/S0140-6736(04)16722-6 15276393

[B31] WHO Regional Office for Europe Who Regional Strategy on Sexual and Reproductive Health 2001 http://www.euro.who.int/__data/assets/pdf_file/0004/69529/e74558.pdf pdf last accessed 17.03.2011

[B32] Bundeszentrale für gesundheitliche Aufklärung **Country papers on youth sexuality in Europe - Synopsis** 2006 BZgA

[B33] Bobrova N Sergeev O Grechukhina T Kapiga S Social-cognitive predictors of consistent condom use among young people in Moscow Perspect Sex Reprod Health 2005 37 4 174 178 10.1363/3717405 16380362

[B34] European Commission Compulsory education in Europe 2010/2011 http://eacea.ec.europa.eu/education/eurydice/documents/compulsory_education/compulsory_education.pdf pdf last accessed 10.05.2011

[B35] Assessing scientific admissibility and merit of published articles: Critical appraisal form http://peds.stanford.edu/Tools/documents/Critical_Appraisal_Form_CGP.pdf last accessed 08.03.2011

[B36] Fogarty J Knowledge about AIDS among leaving certificate students Irish Med Journal 1990 83 19 21 2361831

[B37] Andersson-Ellström A Forssman L Sexually transmitted diseases - knowledge and attitudes among young people J Adolesc Health 1991 12 72 76 10.1016/0197-0070(91)90446-S 2015244

[B38] Tyden T Norden L Ruusuvaara L Swedish students' knowledge of sexually transmitted diseases and their attitudes to the condom Midwifery 1991 7 25 30 10.1016/S0266-6138(05)80131-7 2011089

[B39] Lunin I Hall TL Mandel JS Adolescent sexuality in Saint Petersburg, Russia AIDS 1995 9 suppl 1 S53 S60 8562001

[B40] Andersson-Ellström A Forssman L Milsom I The relationship between knowledge about sexually transmitted diseases and actual sexual behaviour in a group of teenage girls Genitourin Med 1996 72 32 36 8655164 10.1136/sti.72.1.32 PMC1195588

[B41] Eriksson T Sonesson A Isacsson A HIV/AIDS - information and knowledge: a comparative stud of Kenyan and Swedish teenagers Scand J Soc Med 1997 25 111 118 9232721 10.1177/140349489702500208

[B42] Garside R Ayres R Owen M Pearson VAH Roizen J 'They never tell you about the consequences': young people's awareness of sexually transmitted infections Int J STD & AIDS 2001 12 582 588 10.1258/0956462011923750 11516367

[B43] Goodwin R Kozlova A Nizharadze G Polyakove G HIV/AIDS among adolescents in Eastern Euorpe: knowledge of HIV/AIDS, social representations of risk and sexual activity among school children and homeless adolescents in Russia, Georgia and the Ukraine J Health Psych 2004 9 381 396 10.1177/1359105304042348 15117538

[B44] Macek M Matkovic V Attitudes of school environment towards integration of HIV-positive pupils into regular classes and knowledge about HIV/AIDS: cross-sectional study Croat Med J 2005 26 320 325 15849857

[B45] Woodhall Sc Lehtinen M Verho T Huhtala H Hokkanen M Kosunen E Anticipated acceptance of HPV vaccination at the baseline of implementation: a survey of parental and adolescent knowledge and attitudes in Finland J Adolesc Health 2007 40 466 469 10.1016/j.jadohealth.2007.01.005 17448408

[B46] Gottvall M Larsson M Högkund AT Tydén T High HPV vaccine acceptance despite low awareness among Swqedish upper secondary school students Eur J Contr Repr Health Care 2009 14 399 405 10.3109/13625180903229605 19929642

[B47] Höglund AT Tydén T Hannerfors AK Larsson M Knowledge of human papillomavirus and attitudes to vaccination among Swedish high school students Int J STD & AIDS 2009 20 102 107 10.1258/ijsa.2008.008200 19182055

[B48] Pelucchi C Esposito S Galeone C Semino M Sabatini C Picciolli I Consolo S Milani G Principi N Knowledge of human papillomavirus infection and its prevention among adolescents and parents in the greater Milan area, Northern Italy BMC Public Health 2010 10 378 10.1186/1471-2458-10-378 20584324 PMC2901377

[B49] Sachsenweger M Kundt G Hauk G Lafrenz M Stoll R Knowledge of school pupils about the HIV/AIDS topic at selected schools in Mecklenburg-Pomerania: Results of a survey of school pupils Gesundheitswesen 2010, online publication 2.3.2010 http://dx.doi.org/10.1055/s-0029-1246199 10.1055/s-0029-1246199 20198565

[B50] Vinten G Open versus closed questions - an open issue? Manag 1995 33 27 31

[B51] Krosnick JA Presser S Wright JD, Marsden PV Question and Questionnaire Design Handbook of Survey research 2010 2 Bingley: Emerald Group Publishing Ltd 263 314 http://comm.stanford.edu/faculty/krosnick/Handbook%20of%20Survey%20Research.pdf Last accessed 02.05.2011

[B52] Piccinino LJ Mosher WD Trends in contraceptive use in the United States: 1982-1995 Family Planning Perspectives 1998 30 4 10 10.2307/2991517 9494809

[B53] Glei DA Measuring contraceptive use patterns among teenage and adult women Family Planning Perspectives 1999 31 73 80 10.2307/2991642 10224545

[B54] Everett SA Warren CW Santelli JS Kann L Collins JL Kolbe LJ Use of birth control pills, condoms and withdrawal among U.S. high school students Journal of Adolescent Health 2000 27 112 118 10.1016/S1054-139X(99)00125-1 10899471

[B55] Kaaya SF Flisher AJ Mbwambo JK Schaalma H Aaro LE Klepp KI A review of studies of sexual behaviour of school students in sub-Saharan Africa Scandinavian Journal of Public Health 2002 30 148 160 12028864 10.1080/14034940210133807

[B56] Abraham C Wight D Developing HIV-preventive behavioural interventions for young people in Scotland Int Journal of STD and AIDS 1996 7 suppl 2 39 42 10.1258/0956462961917762 8799793

[B57] Helfferich C Heidtke B Country papers on youth sex education in Europe 2006 BZgA

