# Supplementary material for: Awareness and knowledge of sexually transmitted diseases (STDs) among school-going adolescents in Europe: a systematic review of published literature
Source: BMC Public Health. 2011 Sep 25;11:727. doi: 10.1186/1471-2458-11-727 (PMC3189891; doi:10.1186/1471-2458-11-727)
Supplement: Additional file 1 — Review Protocol: The preparation process for the systematic review is documented in the file. Included are the objectives of the review, inclusion and exclusion criteria, the search strategy, definition of outcomes, as well as the data abstraction table. [file 1471-2458-11-727-S1.DOC]

Awareness and knowledge of Sexually Transmitted Diseases (STDs) among school-going adolescents in Europe: A systematic review of published literature.

**Background**

Sexually transmitted diseases (STDs) are a major health problem affecting mostly young people, not only in developing, but also in developed countries. If untreated, sexually transmitted diseases can lead to serious complications and long term consequences in women such as pelvic inflammatory diseases, infertility and cervical cancer.[1] According to data from the World Health Organisation (WHO), an estimated 340 million new cases of curable STDs (gonorrhoea, chlamydia, syphilis and trichomoniasis) occurred world-wide in 1999 in men and women aged 15 – 49 years. About 60% of all persons affected are younger than 35 years of age, and 30% of the cases are younger than 20 years.[1] Between the ages of 14 and 19 years, the number of females affected is double that of males.

In their paper published in 2000, Panchaud et al reported a general decrease of gonorrhoea, syphilis and chlamydia in developed countries both in the general population and among adolescents over the period 1985-1996.[2] From the mid-1990s however, increases in the diagnoses of sexually transmitted infections, in particular syphilis, gonorrhoea and Chlamydia have been reported in several European countries.[3-7] The number of diagnosed gonorrhoea cases at clinics for sexually transmitted infections in England and Wales was said to have risen by 102% during this period; from 10 204 to 20 663 cases. The highest increases were noted among teenagers aged 16-19 years (178% for male and 133% for female patients).[6,8] Increases in gonorrhoea cases were also reported for France, 92% from 1997 to 1998, and for Sweden, 154% from 1995 to 2000. Increases have also been reported for syphilis, an infection which had basically disappeared in many EU countries in the late 1980s and mid 1990s, especially among men who have sex with men.[6]

The problem with most STIs is that they can occur symptom-free and can thus be passed on unaware. On an individual level, their complications can lead to pelvic inflammatory diseases and possibly to ectopic pregnancies and infertility.[9-11] It has also been suggested that the increase in gonorrhoea and chlamydia infections could be linked to the declining fertility rates in Europe. In addition, the treatment costs of PID are considerably high, with its direct and indirect costs in the US being estimated at more than $4.2 billion in 1990 and at almost $17 billion in 1994.[12,13]

The declining age of first sexual intercourse has been proffered as one possible explanation for the increase in numbers of STDs.[7] According to data from different European countries, the average age of first sexual intercourse has decreased over the last three decades, with increasing proportions of adolescents reporting sexual activity before the age of 16 years.[14-19]

An early onset of sexual activity not only increases the probability of having various sexual partners, it also increases the chances of contracting a sexually transmitted infection.[20]

**Objectives of the review**

To try and determine and summarise what European adolescents of school-going age know about sexually transmitted infections

*Secondary objectives*

If possible, the following questions will be answered:

Are there differences in knowledge and awareness by gender and age?

Are there changes in knowledge and awareness over time?

How do adolescents rate their own risk of contracting an STD?

**Methods**

**Criteria for inclusion and exclusion of studies in the review.**

**Inclusion criteria – Study types**: cross-sectional studies

**Inclusion criteria – Study language**: studies should be published in English or German

**Inclusion criteria – Time and Place**: studies conducted in Europe and published between 1990 and 2010

**Inclusion criteria – Study participants**: school attending pupils

**Inclusion criteria – Study focus**: the studies should mainly focus on knowledge and awareness of STDs among school pupils.

**Inclusion criteria – Study outcomes**: the studies included must contain some measurement of knowledge and/awareness. Examples of outcomes may include the following:

- number/proportion of pupils able to identify a particular STD
- number/proportion of pupils aware of modes of transmission of particular infections
- number/proportion of pupils aware that condoms can help prevent transmission of STDs

**Exclusion criteria:**

- case reports
- review, editorials, letters, expert opinions
- studies mainly on sexual activity/behaviour
- studies evaluating intervention programmes
- not specific risk groups (drug users, gay men)

**Search Strategy**

Relevant literature published between 1990 and 2010 will be searched for in PubMed using various combinations of the terms “STD”, “HIV”, “HPV”, “chlamydia”, “syphilis”, “gonorrhoea”, “herpes”, “hepatitis B”, “knowledge”, “awareness”, and “adolescents”.

The exact search terms and the number of articles retrieved at each stage will be noted. Details of retrieved articles will be recorded in a Reference Manager database. Once all searches are completed, titles and abstracts of publications found will be perused by one researcher and those that are obviously unsuitable can be excluded. The decision to include or exclude the remaining articles will only be made once the article has been read. Two researchers will be involved in the selection of relevant articles. References of selected articles will be perused for publications of interest which might have not have been found during the electronic search. Results of the selection procedure will be documented in a flow diagram (see Fig. 1).

**Figure 1: Flow diagram of study selection procedure**

Records found via PubMed search

(n = xx)

Excluded after initial evaluation with reasons

(n = xx)

Full-text articles assessed for eligibility

(n = xx)

Excluded after full evaluation of text with reasons

(n = xx)

Articles selected for review

(n = xx)

Further articles identified from perusal of reference lists of articles selected for review

(n = xx)

Total articles included in review

(n = xx)

**Definition of outcomes**

**Awareness**: able to identify disease as STD either from a given list or in response to an open question.

**Knowledge**: able to identify disease as STD + at least one of the following

- knowledge of modes of transmission
- knowledge of protection
- knowledge of treatment
- knowledge that STD can be symptom-free
- knowledge of places to go for diagnosis/ treatment/ advice

**Data abstraction:**

Two researchers will independently abstract data using Table 1. They will compare the summaries and will consult the original publication to resolve any differences.

**Assessment of methodological quality:**

The methodologies of studies included in the review will be assessed using a modified version of the Critical Appraisal Form from the Stanford School of Medicine as shown in Table 2. The extracted data will be stored in an excel databank.

**Synthesis**

As we don’t expect the studies we retrieve to be homogeneous in nature, we will initially describe how they were conducted and tabulate their respective findings. Meta-analysis methods will only be used if feasible.

**Conflict of interests**

We are unaware of any potential conflict of interests.

Table 1: Data abstraction table

| Study N° |  |
| --- | --- |
| Authors & title |  |
| Study design |  |
| Methods/measures |  |
| Geographic location |  |
| Population included |  |
| Outcome |  |
| Results |  |
| Basic knowledge |  |
| Awareness of STD in general |  |
| Awareness of HPV |  |
| Awareness of HIV |  |
| Awareness of chlamydia |  |
| Awareness of syphilis |  |
| Awareness of gonorrhoea |  |
| Awareness of herpes |  |
| Awareness of hepatitis B |  |
| Further knowledge |  |
| Knowledge that STD can be symptom-free |  |
| Knowledge of modes of transmission |  |
| Knowledge of protection |  |
| Knowledge of treatment |  |
| Knowledge of places to go for diagnosis/ treatment/ advice |  |
| Subjective rating of risk of contracting an STI |  |
| Further concerns/ topics mentioned |  |
| Limitations of study/ comments |  |

Table 2: Critical appraisal form for methodological assessment of studies included in the review

| Criteria | Number of studies in each assessment category* | | | | | | | | |
| --- | --- | --- | --- | --- | --- | --- | --- | --- | --- |
|  | Y | S | N | NC | NR | PR | NA | NP | P |
| Did the study address a clearly focused issue? |  |  |  |  |  |  |  |  |  |
| Was/were the study outcome(s) to be measured clearly described? |  |  |  |  |  |  |  |  |  |
| Were the questions posed to assess outcome(s) clearly defined? |  |  |  |  |  |  |  |  |  |
| Was the study sample clearly defined? |  |  |  |  |  |  |  |  |  |
| Were participating schools recruited in an acceptable way? |  |  |  |  |  |  |  |  |  |
| Were the pupils recruited in an acceptable way? ^1^ |  |  |  |  |  |  |  |  |  |
| Were characteristics of subjects at enrolment reported? |  |  |  |  |  |  |  |  |  |
| Is it clear how data were collected? |  |  |  |  |  |  |  |  |  |
| Did the authors mention that the instrument used for data collection was pre-tested or validated? |  |  |  |  |  |  |  |  |  |
| Were the questions posed appropriate to address given outcomes? |  |  |  |  |  |  |  |  |  |
| Was participation rate reported? |  |  |  |  |  |  |  |  |  |
| Was participation rate sufficiently high? |  |  |  |  |  |  |  |  |  |
| Was the data analysis sufficiently rigorous? |  |  |  |  |  |  |  |  |  |
| Were other factors accounted for that could affect outcomes?^2^ |  |  |  |  |  |  |  |  |  |
| Were results appropriately reported? ^3^ |  |  |  |  |  |  |  |  |  |
| Is there a clear statement of findings? |  |  |  |  |  |  |  |  |  |

*Y= Yes, S= Substandard, N= No, NC= Not Clear, NR= Not Reported, PR= Partially Reported, NA= Not Applicable, NP= Not Possible to Assess, P= Partly

^1^ did all pupils at the school(s), respectively in the grade concerned, have the same chance to participate?

^2^ for example, sex, age, grade, school type, social class

^3^ were numbers of outcome events reported on?

References

1. World Health Organisation: **Global prevalence and incidence of selected curable sexually transmitted infections.** WHO, Geneva, 2001
2. Panchaud C, Singh S, Feivelson D, Darroch JE: **Sexually transmitted diseases among adolescents in developed countries.** *Fam Plan Persp* 2000, **32:** 24-32 & 45
3. Berglund T, Fredlund H, Giesecke J: **Epidemiology of the re-emergence of gonorrhoea in Sweden.** *Sex Transm Dis* 2001, 111-114
4. Health protection Surveillance Centre. **Surveillance of STI.** *A report by the Sexually Transmitted Infections subcommittee for the Scientific Advisory committee of the health Protection Surveillance Centre*, December 2005
5. Nicoll A, Hamers FF: **Are trends in HIV, gonorrhoea and syphilis worsening in Western Europe?** *BMJ* 2002; **324:** 1324-1327
6. Twisselmann B: **Rising trends of HIV, gonorrhoea, and syphilis in Europe make case for introducing European surveillance systems.** *Euro Surveill* 2002, **6** (23): pii=1952 [<http://www.eurosurveillance.org/ViewArticle.aspx?ArticleId=1952>] last accessed 30.11.2010
7. Adler MW: **Sexually transmitted infections in Europe**. *Eurohealth* 2006, **12:** 3-6
8. PHLS, DHSS & PS and the Scottish ISD(D)5 Collaborative Group: **Trends in Sexually Transmitted Infections in the United Kingdom 1990-1999**. Public Health Laboratory Service London; 2000.
9. Stamm W, Guinan M, Johnson C: **Effect of treatment regiments for Neisserie gonorrhoea on simultaneous infections with Chlamydia trachomatis**. *New Eng J Med* 1984, **310:** 545-559
10. MacDonald NE, Brunham R. **The Effects of Undetected and Untreated Sexually Transmitted Diseases: Pelvic Inflammatory Disease and Ectopic Pregnancy in Canada.** *The Canadian Journal of Human Sexuality* 1997, **6 (2) Special Issue: STDs and Sexual/Reproductive Health**
11. Simms I, Stephenson JM: **Pelvic inflammatory disease epidemiology; What do we know and what do we need to know?** *Sex Trans Inf* 2000, **76:** 80-87
12. **Washington A, Katz P: Cost of and payment source for pelvic inflammatory disease. Journal of the American Medical Association 1991; 226: 2565-2569**
13. **Centre of Diseases Control and Prevention. Tracking the hidden epidemics: Trends in STDs in the United States 2000 [**[http://www.cdc.gov/std/trends2000/Trends2000.pdf]](http://www.cdc.gov/std/trends2000/Trends2000.pdf%5D )

last accessed 14.03.2011

1. Kangas I, Andersen B, McGarrigle CA, Ostergaad L: **A comparison of sexual behaviour and attitudes of healthy adolescents in a Danish high school in 1982, 1996 and 2001.** *Pop Health Metr* 2004, online publication: [http://www.pophealthmetrics.com/content/2/1/5] last accessed 03.12.2010
2. **Ross J, Godeau E, Dias S. Sexual health. In *Young people’s health in context. Health Behaviour in School-aged Children (HSBC) study: International report from the 2001/2002 survey*. Edited by Currie C, Roberts C, Morgan A, et al. Copenhagen: WHO; 2004**
3. Bundeszentrale für gesundheitliche Aufklärung: **Jugendsexualität.** **Repräsentative Wiederholungsbefragung von 14- bis 17-Jährigen Jugendlichen und ihren Eltern**. BZgA, 2006.
4. Tucker JS, Fitzmaurice AE, Imamura M, Penfold S, Penney GC, van Teijlingen E, Schucksmith J, Philip KL: **The effect of the national demonstration project *Healthy Respect* on teenage sexual health behaviour.** *Eur J Public Health* 2006, **17** (1): 33-41
5. Godeau E, Gabhainn SN, Vignes C, Ross J, Boyce W, Todd J: **Contraceptive use by 15-year-old students at their last sexual intercourse: Results from 24 countries**. *Arch Paediatr Adolesc Med* 2008, **162**: 66-73
6. Bundeszentrale für gesundheitliche Aufklärung: **Sexualität und Migration: Milieuspezifische Zugangswege für die Sexualaufklärung Jugendlicher. Ergebnisse einer repräsentativen Untersuchung der Lebenswelten von 14- bis 17-Jährigen Jugendlichen mit Migrationshintergrund.** BZgA, 2010.
7. Heinz M: **Sexuell übertragbare Krankheiten bei Jugendlichen: Epidemiologische Veränderungen und neue diagnostische Methoden.** Arbeitsgemeinschaft Kinder-und Jugendgynäkologie e.V. 2001: [http://www.kindergynaekologie.de/html/kora22.html]

last accessed 03.12.2010
